# 1-(Pyrrolidin-1-yl)ethan-1-iminium chloride

**DOI:** 10.1107/S2414314623007903

**Published:** 2023-09-12

**Authors:** Rylan Artis, Clifford W. Padgett, Brandon Quillian

**Affiliations:** a Department of Biochemistry, Chemistry and Physics, Georgia Southern University, Armstrong Campus, 11935 Abercorn Street, Savannah GA 31419, USA; University of Aberdeen, United Kingdom

**Keywords:** aceto­nitrile, amidinium salt, chloro­form, crystal structure, Pinner reaction, pyrrolidine

## Abstract

The title compound, C_6_H_13_N_2_^+^·Cl^−^, is as an amidinium salt that was isolated as unexpected product from the reaction between aceto­nitrile, chloro­form and pyrrolidine under refluxing conditions. The packing features two N—H⋯Cl hydrogen bonds to generate centrosymmetric tetra­mers (two cations and two anions) and van der Waals inter­actions.

## Abstract 2

The title salt was serendipitously obtained while using a mixed solvent system during an S_N_2 reaction between an alkyl halide and pyrrolidine.

## Graphical abstract

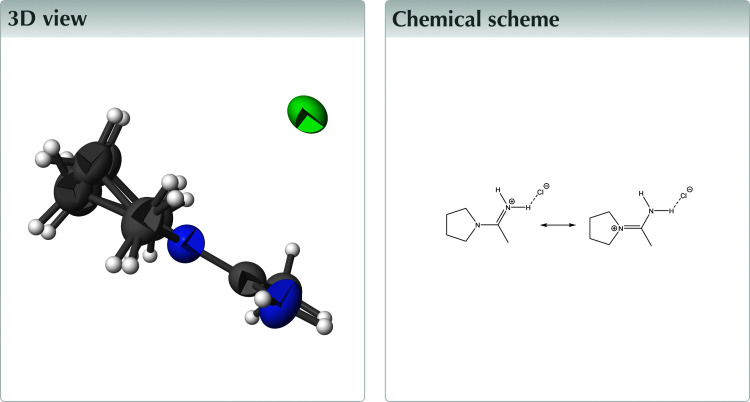

## Structure description

Amidinium salts are protonated amidine compounds characterized by a central carbon atom bound to a protonated imine (iminium) group and a neutral amine. They were first prepared by reacting a Pinner salt with an amine (Pinner & Klein, 1877 ▸). Although acetamidinium salts are generally unstable, an acetamidinium chloride salt was reported in 1976 (Cannon *et al.*, 1976 ▸). This salt has been exploited for its strong hydrogen-bonding properties in subsequent research (Ferretti *et al.*, 2004 ▸; Norrestam, 1984 ▸; Yang *et al.*, 2022 ▸). It has been observed as a counter-ion for anionic transition/main-group metal complexes and perovskites (Liu *et al.*, 2018 ▸; Singh *et al.*, 2021 ▸; Biller *et al.*, 2002 ▸). Amidinium salts derived from alkyl­ated and cyclic amines exhibit greater stability and have also been observed as counter-ions for transition-metal complexes (Podjed & Modec, 2023 ▸).

**Figure 1:**
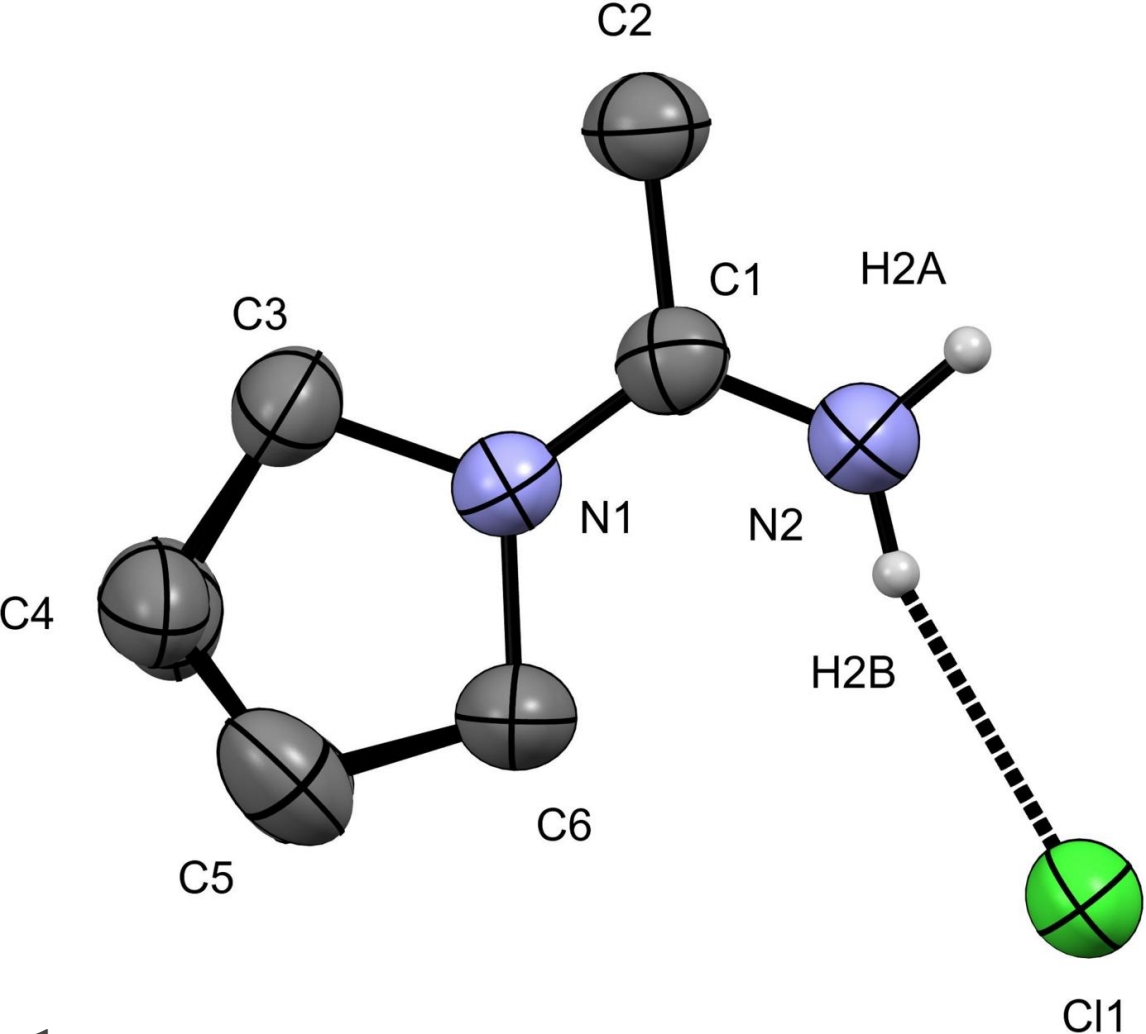
The mol­ecular structure of the title compound (**1**) in the asymmetric unit with displacement ellipsoids drawn at 50%. Hydrogen atoms are removed from carbon atoms for clarity and only the major disorder component is shown.

In regards to the cation in the title compound, C_6_H_13_N_2_^+^·Cl^−^, (**1**), it has mainly been observed in transition and rare-earth metal complexes (Podjed *et al.*, 2020 ▸; Masci & Thuéry, 2003 ▸; Podjed & Modec, 2022 ▸). A piperidine amidinium chloride salt has been reported (Podjed & Modec, 2023 ▸). Herein, we report the structure (Fig. 1 ▸) of the title compound, which crystallizes in the monoclinic crystal system in space group *P*2_1_/*c*. The carbon atoms of the pyrrolo­dine ring are disordered over two sets of sites in a 0.590 (11):0.410 (11) ratio with both disorder components leading to a twisted conformation of the ring.

**Figure 2:**
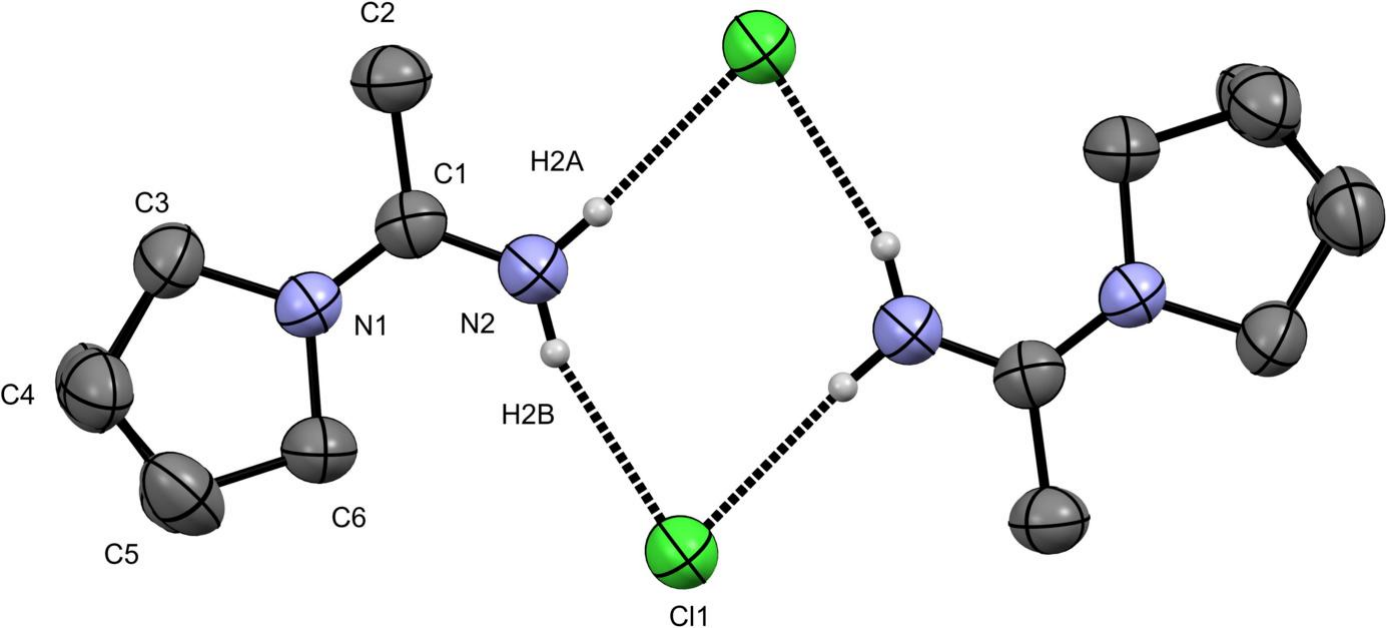
The mol­ecular structure of the dimer of the title compound, showing the hydrogen-bonding network between the NH_2_ group and chloride anion. Displacement ellipsoids are drawn at 50% and hydrogen atoms have been removed from carbon atoms for clarity.

**Figure 3:**
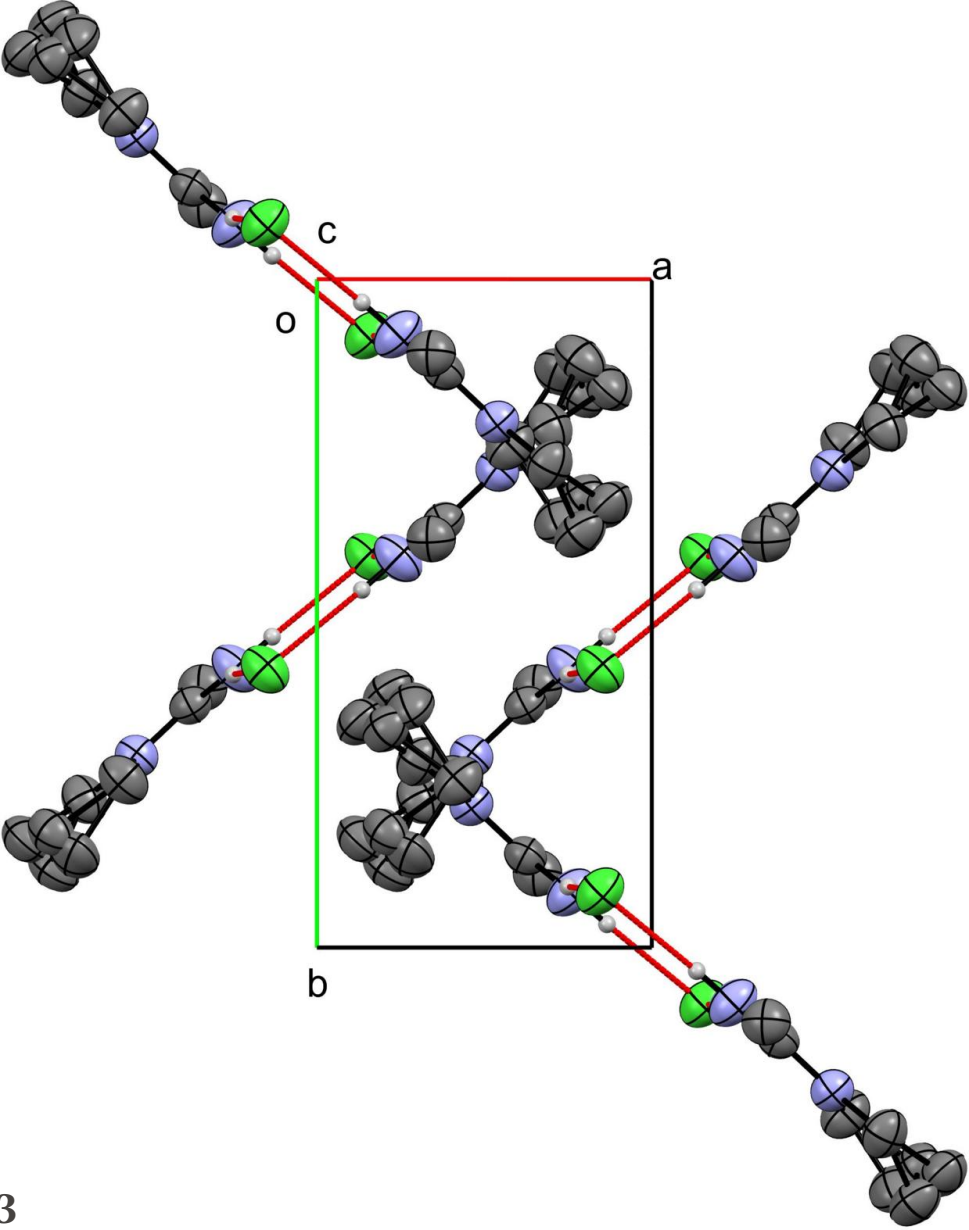
Packing of compound (**1**) viewed along the *c* axis.

In the extended structure of (**1**), a pair of amidinium cations are hydrogen bonded to two chloride ions (Table 1 ▸) forming a hydrogen-bonded tetra­mer (two cations and two anions) with graph set 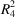 (8) as shown in Fig. 2 ▸. The tetra­mer forms a square with a N⋯Cl ⋯N⋯Cl dihedral angle of 0.00 (8)°. The packing is shown in Fig. 3 ▸. This structural motif closely resembles that of 1-(piperidin-1-yl)ethan-1-iminium chloride (pipim Cl) as reported by Podjed & Modec (2023 ▸). However, the N⋯Cl hydrogen-bond distances in (**1**) (mean = 3.211 Å) are slightly longer than those in pipim Cl, which measure 3.183 Å. Additionally, the C—N bond distances in (**1**) are slightly shorter than those of pipim Cl: in (**1**), C1—N1 is 1.311 (2) Å and C1—N2 is 1.310 (2) Å, while in pipim Cl, they are 1.321 (2) and 1.317 (2) Å, respectively. The geometries at C1 and N1 are nearly perfectly trigonal planar, with a sum of the bond angles around each atom equaling 360.1 and 359.9°, respectively, which are within the expected margin of error.

## Synthesis and crystallization

Pyrrolidine (325 µl, 0.251 g, 3.96 mmol), aceto­nitrile (5 ml, 3.93 g, 96.5 mmol) and chloro­form (1.5 ml, 2.24 g, 18.8 mmol) were combined in a pressure tube. A stir bar was added, and the tube was capped. The mixture was then heated with stirring at 70°C for 8 days. After cooling to room temperature, colorless needle-like crystals formed, yielding 305.6 mg (52%) of the title compound.

## Refinement

Crystal data, data collection and structure refinement details are summarized in Table 2 ▸

## Supplementary Material

Crystal structure: contains datablock(s) I. DOI: 10.1107/S2414314623007903/hb4444sup1.cif

Structure factors: contains datablock(s) I. DOI: 10.1107/S2414314623007903/hb4444Isup2.hkl

Supporting information file. DOI: 10.1107/S2414314623007903/hb4444Isup3.cml

CCDC reference: 2293948

Additional supporting information:  crystallographic information; 3D view; checkCIF report

## Figures and Tables

**Table 1:** Hydrogen-bond geometry (Å, °)

*D*—H⋯*A*	*D*—H	H⋯*A*	*D*⋯*A*	*D*—H⋯*A*
N2—H2*A*⋯Cl1^i^	0.87 (2)	2.33 (2)	3.1988 (16)	175 (2)
N2—H2*B*⋯Cl1^ii^	0.88 (2)	2.38 (2)	3.2230 (16)	162 (2)

Symmetry codes: (i) 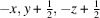 ; (ii) 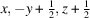 .

**Table 2:** Experimental details

Crystal data	
Chemical formula	C_6_H_13_N_2_^+^·Cl^−^
*M*_r_	148.63
Crystal system, space group	Monoclinic, *P*2_1_/*c*
Temperature (K)	300
*a*, *b*, *c* (Å)	5.7234 (1), 11.2961 (1), 12.6591 (2)
β (°)	98.820 (1)
*V* (Å^3^)	808.76 (2)
*Z*	4
Radiation type	Cu *K*α
μ (mm^−1^)	3.53
Crystal size (mm)	0.3 × 0.1 × 0.1

Data collection	
Diffractometer	XtaLAB Synergy, Single source at home/near, HyPix3000
Absorption correction	Multi-scan (*CrysAlis PRO*; Rigaku OD, 2023 ▸)
*T*_min_, *T*_max_	0.326, 1.000
No. of measured, independent and observed [*I* > 2σ(*I*)] reflections	8326, 1514, 1343
*R*_int_	0.034
(sin θ/λ)_max_ (Å^−1^)	0.608

Refinement	
*R*[*F* ^2^ > 2σ(*F* ^2^)], *wR*(*F* ^2^), *S*	0.033, 0.100, 1.09
No. of reflections	1514
No. of parameters	111
No. of restraints	7
H-atom treatment	H atoms treated by a mixture of independent and constrained refinement
Δρ_max_, Δρ_min_ (e Å^−3^)	0.20, −0.18

Computer programs: *CrysAlis PRO* (Rigaku OD, 2023 ▸), *SHELXT2018/2* (Sheldrick, 2015*a* ▸), *SHELXL2018/3* (Sheldrick, 2015*b* ▸), and *OLEX2* (Dolomanov *et al.*, 2009 ▸).

## full crystallographic data

### Crystal data

**Table:**

C_6_H_13_N_2_^+^·Cl^−^	*F*(000) = 320
*M**_r_* = 148.63	*D*_x_ = 1.221 Mg m^−^^3^
Monoclinic, *P*2_1_/*c*	Cu *K*α radiation, λ = 1.54184 Å
*a* = 5.7234 (1) Å	Cell parameters from 5627 reflections
*b* = 11.2961 (1) Å	θ = 3.5–69.3°
*c* = 12.6591 (2) Å	µ = 3.53 mm^−^^1^
β = 98.820 (1)°	*T* = 300 K
*V* = 808.76 (2) Å^3^	Needle, clear light yellow
*Z* = 4	0.3 × 0.1 × 0.1 mm

### Data collection

**Table:**

XtaLAB Synergy, Single source at home/near, HyPix3000 diffractometer	1514 independent reflections
Radiation source: micro-focus sealed X-ray tube, PhotonJet (Cu) X-ray Source	1343 reflections with *I* > 2σ(*I*)
Mirror monochromator	*R*_int_ = 0.034
Detector resolution: 10.0000 pixels mm^-1^	θ_max_ = 69.8°, θ_min_ = 5.3°
ω scans	*h* = −6→6
Absorption correction: multi-scan (CrysAlisPro; Rigaku OD, 2023)	*k* = −13→13
*T*_min_ = 0.326, *T*_max_ = 1.000	*l* = −15→15
8326 measured reflections	

### Refinement

**Table:**

Refinement on *F*^2^	Hydrogen site location: mixed
Least-squares matrix: full	H atoms treated by a mixture of independent and constrained refinement
*R*[*F*^2^ > 2σ(*F*^2^)] = 0.033	*w* = 1/[σ^2^(*F*_o_^2^) + (0.0521*P*)^2^ + 0.1032*P*] where *P* = (*F*_o_^2^ + 2*F*_c_^2^)/3
*wR*(*F*^2^) = 0.100	(Δ/σ)_max_ < 0.001
*S* = 1.09	Δρ_max_ = 0.20 e Å^−^^3^
1514 reflections	Δρ_min_ = −0.17 e Å^−^^3^
111 parameters	Extinction correction: SHELXL-2018/3 (Sheldrick 2015b), Fc^*^=kFc[1+0.001xFc^2^λ^3^/sin(2θ)]^-1/4^
7 restraints	Extinction coefficient: 0.0060 (11)
Primary atom site location: dual	

### Special details

**Table:**

Geometry. All esds (except the esd in the dihedral angle between two l.s. planes) are estimated using the full covariance matrix. The cell esds are taken into account individually in the estimation of esds in distances, angles and torsion angles; correlations between esds in cell parameters are only used when they are defined by crystal symmetry. An approximate (isotropic) treatment of cell esds is used for estimating esds involving l.s. planes.

### Fractional atomic coordinates and isotropic or equivalent isotropic displacement parameters (Å^2^)

**Table:**

	*x*	*y*	*z*	*U*_iso_*/*U*_eq_	Occ. (<1)
N1	0.5387 (2)	0.28640 (11)	0.42307 (9)	0.0501 (3)	
N2	0.2421 (3)	0.41737 (13)	0.44168 (12)	0.0639 (4)	
C1	0.3772 (3)	0.36286 (13)	0.38250 (12)	0.0493 (4)	
C2	0.3413 (3)	0.38872 (15)	0.26616 (13)	0.0610 (4)	
H2C	0.485848	0.418222	0.246373	0.091*	
H2D	0.219266	0.447140	0.249911	0.091*	
H2E	0.295598	0.317535	0.226955	0.091*	
C3	0.6948 (3)	0.22165 (16)	0.36067 (14)	0.0637 (4)	
H3AA	0.823737	0.271321	0.345347	0.076*	0.590 (11)
H3AB	0.607714	0.192420	0.293987	0.076*	0.590 (11)
H3BC	0.774587	0.275324	0.318169	0.076*	0.410 (11)
H3BD	0.607101	0.163573	0.313957	0.076*	0.410 (11)
C6	0.5776 (3)	0.25090 (17)	0.53598 (13)	0.0645 (5)	
H6AA	0.442485	0.207796	0.553965	0.077*	0.590 (11)
H6AB	0.606325	0.319377	0.582389	0.077*	0.590 (11)
H6BC	0.429154	0.235374	0.561480	0.077*	0.410 (11)
H6BD	0.664313	0.311035	0.580605	0.077*	0.410 (11)
C4	0.7858 (13)	0.1195 (5)	0.4355 (4)	0.0712 (14)	0.590 (11)
H4A	0.679085	0.052351	0.425390	0.085*	0.590 (11)
H4B	0.941834	0.094355	0.423829	0.085*	0.590 (11)
C5	0.7933 (11)	0.1725 (6)	0.5457 (4)	0.0648 (14)	0.590 (11)
H5A	0.786343	0.111081	0.598645	0.078*	0.590 (11)
H5B	0.936561	0.218358	0.565710	0.078*	0.590 (11)
C4A	0.8700 (14)	0.1621 (8)	0.4477 (6)	0.0741 (19)	0.410 (11)
H4AA	0.930051	0.088867	0.422068	0.089*	0.410 (11)
H4AB	1.001716	0.213995	0.472880	0.089*	0.410 (11)
C5A	0.7221 (17)	0.1389 (8)	0.5339 (8)	0.079 (3)	0.410 (11)
H5AA	0.820851	0.125641	0.602236	0.094*	0.410 (11)
H5AB	0.620598	0.070640	0.516567	0.094*	0.410 (11)
Cl1	0.15495 (7)	0.08401 (4)	0.18724 (3)	0.0659 (2)	
H2A	0.134 (3)	0.4650 (16)	0.4103 (15)	0.074 (6)*	
H2B	0.250 (4)	0.4087 (18)	0.5110 (13)	0.082 (7)*	

### Atomic displacement parameters (Å^2^)

**Table:**

	*U*^11^	*U*^22^	*U*^33^	*U*^12^	*U*^13^	*U*^23^
N1	0.0544 (7)	0.0526 (7)	0.0435 (6)	0.0031 (6)	0.0077 (5)	−0.0016 (5)
N2	0.0708 (10)	0.0699 (9)	0.0509 (8)	0.0214 (7)	0.0092 (7)	0.0035 (7)
C1	0.0526 (8)	0.0470 (8)	0.0477 (8)	−0.0048 (6)	0.0064 (6)	−0.0024 (6)
C2	0.0707 (10)	0.0635 (10)	0.0476 (9)	0.0005 (8)	0.0059 (7)	0.0030 (7)
C3	0.0639 (10)	0.0725 (10)	0.0556 (9)	0.0103 (8)	0.0120 (7)	−0.0083 (8)
C6	0.0732 (11)	0.0722 (11)	0.0474 (8)	0.0148 (8)	0.0077 (8)	0.0055 (7)
C4	0.081 (3)	0.068 (3)	0.063 (2)	0.022 (2)	0.004 (2)	−0.009 (2)
C5	0.066 (3)	0.067 (3)	0.057 (2)	0.011 (2)	−0.0018 (19)	−0.0028 (18)
C4A	0.069 (4)	0.068 (4)	0.084 (4)	0.017 (3)	0.008 (3)	−0.008 (3)
C5A	0.070 (5)	0.068 (5)	0.094 (6)	0.010 (3)	0.003 (4)	0.024 (4)
Cl1	0.0682 (3)	0.0695 (3)	0.0592 (3)	−0.01566 (18)	0.0073 (2)	−0.00212 (17)

### Geometric parameters (Å, º)

**Table:**

N1—C1	1.3107 (18)	C6—H6AA	0.9700
N1—C3	1.4746 (19)	C6—H6AB	0.9700
N1—C6	1.4682 (19)	C6—H6BC	0.9700
N2—C1	1.310 (2)	C6—H6BD	0.9700
N2—H2A	0.870 (15)	C6—C5	1.509 (6)
N2—H2B	0.877 (16)	C6—C5A	1.514 (9)
C1—C2	1.485 (2)	C4—H4A	0.9700
C2—H2C	0.9600	C4—H4B	0.9700
C2—H2D	0.9600	C4—C5	1.512 (6)
C2—H2E	0.9600	C5—H5A	0.9700
C3—H3AA	0.9700	C5—H5B	0.9700
C3—H3AB	0.9700	C4A—H4AA	0.9700
C3—H3BC	0.9700	C4A—H4AB	0.9700
C3—H3BD	0.9700	C4A—C5A	1.503 (10)
C3—C4	1.533 (5)	C5A—H5AA	0.9700
C3—C4A	1.527 (7)	C5A—H5AB	0.9700
			
C1—N1—C3	124.45 (13)	N1—C6—C5A	102.3 (4)
C1—N1—C6	123.65 (13)	H6AA—C6—H6AB	109.0
C6—N1—C3	111.85 (12)	H6BC—C6—H6BD	109.2
C1—N2—H2A	118.2 (13)	C5—C6—H6AA	111.0
C1—N2—H2B	125.1 (14)	C5—C6—H6AB	111.0
H2A—N2—H2B	116.7 (19)	C5A—C6—H6BC	111.3
N1—C1—C2	120.03 (14)	C5A—C6—H6BD	111.3
N2—C1—N1	121.89 (14)	C3—C4—H4A	111.1
N2—C1—C2	118.08 (14)	C3—C4—H4B	111.1
C1—C2—H2C	109.5	H4A—C4—H4B	109.1
C1—C2—H2D	109.5	C5—C4—C3	103.4 (4)
C1—C2—H2E	109.5	C5—C4—H4A	111.1
H2C—C2—H2D	109.5	C5—C4—H4B	111.1
H2C—C2—H2E	109.5	C6—C5—C4	104.5 (4)
H2D—C2—H2E	109.5	C6—C5—H5A	110.9
N1—C3—H3AA	111.2	C6—C5—H5B	110.9
N1—C3—H3AB	111.2	C4—C5—H5A	110.9
N1—C3—H3BC	111.3	C4—C5—H5B	110.9
N1—C3—H3BD	111.3	H5A—C5—H5B	108.9
N1—C3—C4	102.6 (2)	C3—C4A—H4AA	111.2
N1—C3—C4A	102.5 (3)	C3—C4A—H4AB	111.2
H3AA—C3—H3AB	109.2	H4AA—C4A—H4AB	109.1
H3BC—C3—H3BD	109.2	C5A—C4A—C3	102.7 (6)
C4—C3—H3AA	111.2	C5A—C4A—H4AA	111.2
C4—C3—H3AB	111.2	C5A—C4A—H4AB	111.2
C4A—C3—H3BC	111.3	C6—C5A—H5AA	111.0
C4A—C3—H3BD	111.3	C6—C5A—H5AB	111.0
N1—C6—H6AA	111.0	C4A—C5A—C6	103.7 (6)
N1—C6—H6AB	111.0	C4A—C5A—H5AA	111.0
N1—C6—H6BC	111.3	C4A—C5A—H5AB	111.0
N1—C6—H6BD	111.3	H5AA—C5A—H5AB	109.0
N1—C6—C5	103.9 (2)		
			
N1—C3—C4—C5	−31.8 (6)	C3—N1—C1—C2	0.3 (2)
N1—C3—C4A—C5A	32.2 (9)	C3—N1—C6—C5	8.1 (3)
N1—C6—C5—C4	−28.2 (6)	C3—N1—C6—C5A	−13.8 (4)
N1—C6—C5A—C4A	34.0 (9)	C3—C4—C5—C6	37.6 (8)
C1—N1—C3—C4	−162.4 (3)	C3—C4A—C5A—C6	−41.7 (11)
C1—N1—C3—C4A	171.2 (4)	C6—N1—C1—N2	2.5 (2)
C1—N1—C6—C5	−174.6 (3)	C6—N1—C1—C2	−176.66 (15)
C1—N1—C6—C5A	163.5 (4)	C6—N1—C3—C4	14.9 (3)
C3—N1—C1—N2	179.53 (16)	C6—N1—C3—C4A	−11.5 (4)

### Hydrogen-bond geometry (Å, º)

**Table:**

*D*—H···*A*	*D*—H	H···*A*	*D*···*A*	*D*—H···*A*
N2—H2*A*···Cl1^i^	0.87 (2)	2.33 (2)	3.1988 (16)	175 (2)
N2—H2*B*···Cl1^ii^	0.88 (2)	2.38 (2)	3.2230 (16)	162 (2)

Symmetry codes: (i) −*x*, *y*+1/2, −*z*+1/2; (ii) *x*, −*y*+1/2, *z*+1/2.
